# Effects of major air pollutants on cognitive function in middle-aged and elderly adults: Panel data evidence from China Health and Retirement Longitudinal Study

**DOI:** 10.7189/jogh.14.04153

**Published:** 2024-11-08

**Authors:** Yingjie Chen, Yinqiao Dong, Yinghuan Zhang, Danni Xia, Yuxuan Wang, Ying Wang, Yong Cai, Fan Hu

**Affiliations:** 1 Public Health department, International Institute of Medicine, Tongren Hospital, Shanghai Jiao Tong University School of Medicine, Shanghai, PR China; 2 School of Public Health, Shanghai Jiao Tong University School of Medicine, Shanghai, PR China

## Abstract

**Background:**

Although numerous studies have discussed about the impact of air pollution on cognitive function, a consensus has yet to be reached, necessitating further exploration of their relationship. The aim of this study is to reveal the effects of major air pollutants on cognitive function in Chinese middle-aged and older adults, while considering the lagged effects of pollution.

**Methods:**

Panel data were constructed by integrating the air pollutants concentration (particulate matter diameter ≤1 µm (μm) (PM_1_), PM_2.5_, PM_10_, nitrogen dioxide (NO_2_), and ozone (O_3_)) among 28 provinces in China and the personal characteristics from China Health and Retirement Longitudinal Study participants during the period of 2011–2015. To explore the effects of single pollutants and their interactions on cognitive function, panel linear regression using ordinary least squares method was employed, and first-order lag effects (two-year interval) of air pollution were introduced into the models.

**Results:**

Our study revealed that, after adjusting for confounding factors, higher levels of particulate matter (PM_1_, coefficient (Coef.) = −0.093, *P* = 0.001; PM_2.5_, Coef. = −0.051, *P* = 0.001; PM_10_, Coef. = −0.030, *P* = 0.001) and NO_2_ (Coef. = −0.094, *P* = 0.006) were associated with lower cognitive function scores among the participants. Moreover, the interaction between the five major pollutants exhibited a negative effect on cognitive function(Coef. = −2.89, *P* = 0.004).

**Conclusions:**

PM_1_, PM_2.5_, PM_10_ have detrimental effects on the cognitive function of middle-aged and elderly adults in China, where increasing particle diameter correlates with a less negative impacts, providing theoretical underpinnings for the formulation of environmental protection policies.

The rapid global population aging has raised concerns regarding the well-being and life quality of elderly adults [1]. This demographic shift is accompanied by a well-documented age-related decline in cognitive function, as widely studied by researchers [2,3], which manifests in various changes in neurological function, including impairments in episodic memory, attention, orientation, and visuo-construction. Importantly, these cognitive changes may have significant implications for the well-being and daily functioning of individuals in later life [4–6]. Cognitive decline represents a transitional state between normal aging and dementia, crucial for the early diagnosis and prediction of mild cognitive impairment (MCI) and dementia [7]. With the anticipated sharp rise in the absolute number of individuals living with dementia [8], there will be substantial strain on public health care systems, particularly in developing countries where aging is rapidly increasing and relative risk (RR) to death is higher (RR = 2.77; 95% confidence interval (CI) = 2.47, 3.10) [9–11].

In addition to aging, cognitive performance varied by gender, geography, education, marital status, and social participation [12]. Studies have indicated that Chinese women are 1.65 times more likely to develop dementia than men [3]. Additionally, lifestyle factors such as smoking, alcohol consumption, as well as overweight and obesity, and chronic diseases have been shown to negatively affect cognitive function [13]. While effective strategies for treating dementia or delaying cognitive decline are currently lacking [14], targeted measures need to be taken to address modifiable factors influencing cognitive performance, according to the recent Lancet Commission on dementia prevention, intervention, and care, where air pollution is declared as one of the modifiable factors [15].

Air pollutant is an umbrella of gaseous pollutants and particulate matter that endanger health. The former covers nitrogen dioxide (NO_2_), ozone (O_3_) et al., and the latter consists of particulate matter (PM) with different aerodynamic diameters, such as those ≤2.5 µm (μm) (diameter ≤2.5μm, PM_2.5_) and ≤10 μm (diameter ≤10 μm, PM_10_) [16]. Particulate matters can easily translocate into the alveolar region from where it reaches to the systemic circulation and into the brain, activate innate immune responses, leading to activation of microglia, and then increase lipid peroxidation and neuroinflammation in various brain regions, particularly the hippocampus and cortex, resulted in causing neuropathy. animal models have correlated exposure to air pollution with oxidative stress, neuroinflammation, cerebrovascular dysfunction, and damage to the blood-brain barrier, leading to impaired cognitive function and increased risk of neurodegenerative diseases [17,18]. Nevertheless, the exact mechanism of air pollution-induced neurotoxicity remains unclear. Epidemiological studies linking air pollution to various endpoints in cognitive decline process have covered the entire life course [19], however, evidence for the middle-aged and elderly population has been less consistent [20–22]. Additionally, the existing reports are mostly limited to cross-sectional studies, and the confounding effects of time-varying variables, such as changes in lifestyle have not been thoroughly investigated so far. Causal inference is also limited, with a majority of studies relying on cross-sectional or cohort designs and employing Bayesian regression analysis to explore associations between mixture exposures and cognitive functioning. There has been a limited application of longitudinal methods, particularly panel data regression, to capture the potential time-varying effects. Therefore, it is important to enhance our understanding of harmful effects of suspected air pollutants on cognitive function, especially enriching the evidence from developing countries, where aging populations and air pollution are exacerbating. Elderly individuals in developing countries may encounter a multitude of challenges, including but not limited to, limited access to medical resources, lower health awareness, and other factors that can adversely affect cognitive functioning. Based on the 2017 Global Burden of Disease study, in China's years of life lost (YLLs) rank, Alzheimer disease and other dementias have surged from 28th place in 1990 to eighth place. Cognitive decline, as a precursor to these neurodegenerative diseases, has become a significant factor threatening the health of the elderly. It urgently requires attention and resolution, to provide insights for many developing countries to address similar issues.

To address this gap, we leveraged a nationwide survey of middle-old aged and elderly people with individual-level air pollution exposure estimates to establish panel data for the years 2011–2015. Our aim was to assess whether air pollution affects cognitive function among Chinese middle-aged and elderly people. Specifically, considering characteristics both at baseline and during the study period, along with air pollution lagged effects, we hypothesised that air pollutants, indicated by the concentrations of PM_1_, PM_2.5_, PM_10_, NO_2_, and O_3_, are negatively associated with the cognitive functioning scores. To validate our hypothesis, panel regression was employed to validate our hypothesis, to examine the independent effects of each air pollutant. Additionally, we explored the role played by pollutants interactions.

## METHODS

### Study design and participants

This study is based on China Health and Retirement Longitudinal Study (CHARLS), an ongoing nationally representative longitudinal survey of middle-aged and older adults that provides high-quality microdata for analysing families and individuals aging in China. The study was approved by the Ethics Committee of Peking University, and each participant provided written informed consent. For complete information on the data provided by CHARLS and precise details of data collection, please refer to their website [23]. To obtain time-interval consistent survey data to construct panel data, we focused on data from three waves conducted in 2011, 2013 and 2015. In CHARLS, the multi-stage stratified sampling approach was implemented at household level. Initially, a certain number of counties were randomly selected from provinces across China. Subsequently, a specific number of villages or urban communities were chosen, and then, households were selected in each village or community. In each sampled household, individuals aged 50 and above were identified for the survey. China Health and Retirement Longitudinal Study encompasses urban and rural areas, as well as different regions, income levels, and age groups among the middle-aged and elderly population to ensure sampling representativeness and generalisability. Trained interviewers conducted face-to-face interviews with participants using a Computer Assisted Personal Interviewing (CAPI) instrument on a personal computer, which facilitated data entry and automated logical skips between survey items based on pre-programmed instructions. Upon completion of the interviews, the Sample Management System (SMS) selected and processed sample records based on predefined criteria. The SMS stores contact information, such as addresses, to maintain participant linkages at the household level. The study was approved by the Ethics Committee of Peking University, and each participant provided written informed consent. For complete information on the data provided by CHARLS and precise details of data collection, please refer to their website. The response rates for these three wave surveys were all above 80%. Participants were required to have demographic, lifestyle, and health status information available for each wave of data collection, along with complete cognitive function assessments, ensuring no missing records. Furthermore, to avoid the bias caused by outliers in body mass index (BMI), systolic blood pressure (SBP), and diastolic blood pressure (DBP), the following criteria were taken for exclusion: participants younger than 45 years, SBP less than 55 or greater than 215, DBP less than 40 or greater than 150, and BMI less than 14 or greater than 45 were excluded.

### Environmental exposure

The assessment of air pollution exposure were based on the China High Pollutant (CHAP) data set [24–28]. China High Pollutant data set is a ground-level air pollutant data source generated through a combination of advanced satellite remote sensing and machine learning algorithms, considering the spatiotemporal heterogeneity of air pollution, with the characteristic of full coverage, high resolution, and high accuracy via cross-validation. It provides concentrations of PM_1_, PM_2.5_, PM_2.5_, O_3_, and NO_2_ in China on a daily basis from 2011–2015 for this study. PM_10_, O_3_, and NO_2_ concentrations, where PM_1_, PM_2.5_, PM_10_, and NO_2_ concentrations are measured using daily averages, while O_3_ is measured using maximum eight-hour averages. For privacy considerations, details about residential addresses were redacted in CHARLS publicly available data, thus, the environmental air pollutant exposure was matched by participant's community ID and the yearly concentrations for each air pollutant were calculated at the city-level averages as a proxy. Moreover, the average pollution concentration for each province were computed based on the corresponding pollutant exposure of individual participants. The spatial distribution maps illustrating the concentrations of the five pollutants in each wave were generated using QGIS.

### Outcomes

In CHARLS, the cognitive functioning was assessed through face-to-face interviews in local languages and this structural questionnaires has been frequently used to track changes in cognitive function over time [29]. The questionnaire contains four dimensions: orientation (respondents were asked about the year, month, day and day of the week on test day: ‘Please tell me today’s date?’ and ‘Please tell me the day of the week. Is it Monday, Tuesday, Wednesday, Thursday, Friday, Saturday, or Sunday?’), visual construction (draw a two pentagons overlapped geometric pattern that shown in the questionnaire), attention (minus 7 five times in a row from 100), and episodic memory (recall 10 Chinese nouns given by the interviewer as many as possible in any order immediately, and recall them again after a while. The number of words successfully repeated is used as the performance of instant memory and delayed memory respectively). Each item is scored 1 point for correct answers, no points for wrong answers or non-answers, and the total score ranges from 0 to 30 points, with a higher the score indicating better cognitive function.

### Covariates

On the basis of our prior knowledge and literature review, a set of variables were recognised to be related to cognitive function: categorical covariates include age (≤60, >60), gender (male, female), education level (illiterate, primary education and secondary education), marital status (married or partnered, single), residence (rural village or urban community), previously diagnosed diseases, including hypertension, diabetes, heart problems, stroke, and dyslipidemia (yes, no), daily behaviours such as drink, smoke and social activity (yes, no), major life challenges like life event and child death (yes, no). SBP (mmHg), DBP (mmHg), and BMI (kg/m^2^), the indicators from physical checkup were considered as continuous covariates. The history of diseases were requested to be diagnosed by professionals and the sociodemographic and lifestyle information were collected by trained interviewer in face-to-face interviews.

### Statistical analysis

Descriptive statistics for panel data, and examined the distribution of all pollutants and variables. The continuous variables are presented as median and inter-quartile range (IQR), and categorical variables were presented as n (%). We used χ^2^ tests and Kruskal-Wallis Rank sum test (K-W test) to assess the characteristics of the participants among years. Pearson correlation analysis was applied to assess the correlations among the five air pollutants in each wave. For each air pollutant concentration across the three waves, we conducted a temporal trend analysis using a polynomial contrast procedure.

We constructed panel databased on unique participant IDs and survey waves (time variables). Next, we established Ordinary Least Squares (OLS) linear regression models, with cognitive functioning scores as the dependent variable. To identify covariates, our study employed univariate and multivariate linear regression model among potential impact factors. The variables showed significant associations with cognitive function were set as covariates. To illustrate the unidirectional causal relationship between air pollution and cognitive function, we incorporated first-order lag effects (two-year interval) to capture the delayed impact of air pollution on the cognitive function of participants, which can effectively take the potential time delay on physical and functional changes into account [30]. Each pollutant concentration, along with the covariates was included in the panel regression models. Furthermore, by incorporating the lagged effects of various pollutants and their interactions in our multivariate regression model, alongside the covariates significantly associated with cognitive function, we constructed a comprehensive panel data linear regression framework. To ensure the reliability of our findings, we employed both default standard errors and White's heteroscedasticity-robust standard errors in building the linear regression models twice.

Besides, to further clarify the relationship between different sections of cognitive function and air pollution exposure, we extended the analysis to include the sections of orientation, attention, visual-construction, and episodic memory based on the established panel data. The same approach was employed for each section.

Since the number of individuals (N) in the short panel data utilised in this study exceeds the number of time periods (T), it is unnecessary to conduct the unit root test. Throughout our panel data analysis, to examine the correlation between individual effects and explanatory variables in the model, Hausman test was employed. The null hypothesis of the test asserts that there is no correlation between individual effects and explanatory variables in the random effects model. A *P*-value below 0.05 indicates that the fixed effects model was suitable, providing evidence to reject the null hypothesis. Otherwise, the random effects model should be adapted. All statistical tests were two-sided, and a *P*-value less than 0.05 was considered statistically significant. All statistical analyses were performed with R software (Version 4.2.0, 2022, Auckland, New Zealand).

## RESULTS

### Basic characteristics and air pollutants distribution

**Figure 1:**
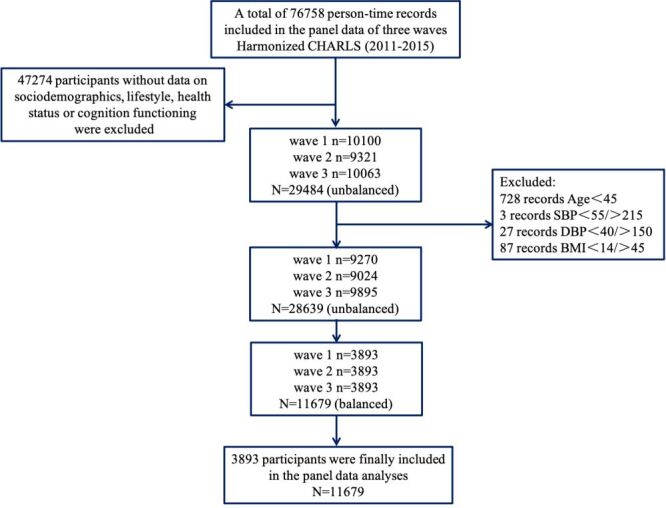
The flowchart of participants selection.

**Figure 2:**
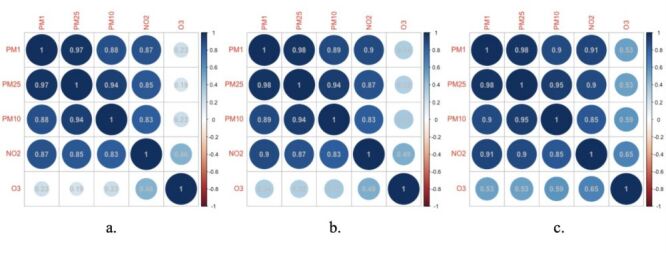
The correlation plots of the five air pollutants. **Panel A.** Wave 1. **Panel B.** Wave 2. **Panel C.** Wave 3.

**Figure 3:**
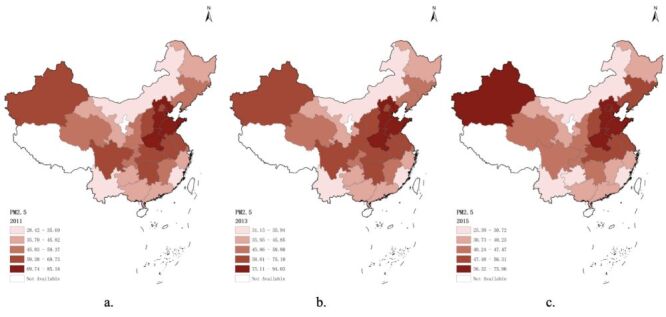
The spatial distribution of average PM_2.5_ concentration. **Panel A.** Wave 1. **Panel B.** Wave 2. **Panel C.** Wave 3. PM – particulate mater

In the three waves from 2011 to 2015, 76 758 observations, 28 856 participants were recorded in panel data exported from Harmonized CHARLS. Three thousand eight hundred ninety-three participants (2095 men and 1798 women) met the inclusion criteria were eventually enrolled in our study, covering 125 cities in 28 provinces in China. The participants enrollment flowchart is shown in **Figure 1** and the basic characteristics of participants and the distributions of the five air pollutants concentrations in each wave were presented in **Table 1**. A majority of our participants were non-illiterate (86.7%) and non-single (90.4%). More than half lived in rural areas (64.3%), reported being socially active (53.7%). The proportion of participants with chronic diseases is less than half, among which hypertension accounts for 28.2%, diabetes 8.02%, heart problems 14.9%, stroke 2.47%, and dyslipidemia 14.7%. Approximately half of them were smokers (46.0%) and consumed alcohol (46.3%). 9.96% participants have experienced child death, and 12.8% have encountered life events. The average blood pressure levels are SBP 125 mm of mercury (mmHg) and DBP 74 mm Hg, and the median BMI is 23.5. Overall, education level and residence remained the same in all three waves; marital status, residence, hypertension, diabetes, heart problems, dyslipidemia, drink, smoke, social work, SBP, BMI, life event, child death were significantly different among waves. There are positive correlations among five air pollutants (**Figure 2**). Additionally, a strong positive correlation among three particulate matters were found (Pearson correlation coefficient (r)>0.9, *P* < 0.05). Besides that, the spatial distribution of air pollutant concentrations is visually depicted in **Figure 3** and Figures S1–4 in the **Online Supplementary Document**. Compared to 2011, the lowest concentration of pollutants in each province decreased in 2015: PM_1_ dropped from 14.82 to 14.80 μg/m^3^, PM_2.5_ dropped from 28.42 to 25.39 μg/m^3^, PM_10_ dropped from 49.90 to 45.66 μg/m^3^, NO_2_ dropped from 16.81 to 16.76 μg/m^3^, and O_3_ dropped from 76.03 to 72.43 μ g/m^3^. The *P*-value in linear trend test for each of the air pollutants was found to be 0.001, indicating that there is a linear trend across different survey times influencing the concentration of air pollutants. The test results were shown in Table S1 in the **Online Supplementary Document**.

**Table 1:** Summary statistics over the entire panel dimension

Characteristics	All (n = 11679)	Wave 1 (n = 3893)	Wave 2 (n = 3893)	Wave 3 (n = 3893)	*P-*value
Gender (n, %)					
*Male*	6285 (53.8)	2095 (53.8)	2095 (53.8)	2095 (53.8)	1.000
*Female*	5394 (46.2)	1798 (46.2)	1798 (46.2)	1798 (46.2)	
Education level (n, %)					
*Illiterate*	1551 (13.3)	517 (13.3)	517 (13.3)	517 (13.3)	1.000
*Primary education*	5409 (46.3)	1803 (46.3)	1803 (46.3)	1803 (46.3)	
*Secondary education and above*	4719 (40.4)	1573 (40.4)	1573 (40.4)	1573 (40.4)	
Marital status (n, %)					
*Married or partnered*	10555 (90.4)	3569 (91.7)	3527 (90.6)	3459 (88.9)	0.001*
*Single*	1124 (9.62)	324 (8.32)	366 (9.40)	434 (11.1)	
Residence (n, %)					
*Rural*	7506 (64.3)	2502 (64.3)	2502 (64.3)	2502 (64.3)	1.000
*Urban*	4173 (35.7)	1391 (35.7)	1391 (35.7)	1391 (35.7)	
Social work (n, %)					
*No*	5403 (46.3)	1927 (49.5)	1607 (41.3)	1869 (48.0)	0.001*
*Yes*	6276 (53.7)	1966 (50.5)	2286 (58.7)	2024 (52.0)	
Drink (n, %)					
*No*	6266 (53.7)	2231 (57.3)	2035 (52.3)	2000 (51.4)	0.001*
*Yes*	5413 (46.3)	1662 (42.7)	1858 (47.7)	1893 (48.6)	
Smoke (n, %)					
*No*	6305 (54.0)	2214 (56.9)	2087 (53.6)	2004 (51.5)	0.001*
*Yes*	5374 (46.0)	1679 (43.1)	1806 (46.4)	1889 (48.5)	
Hypertension (n, %)					
*No*	8384 (71.8)	2953 (75.9)	2824 (72.5)	2607 (67.0)	0.001*
*Yes*	3295 (28.2)	940 (24.1)	1069 (27.5)	1286 (33.0)	
Diabetes (n, %)					
*No*	10742 (92.0)	3651 (93.8)	3591 (92.2)	3500 (89.9)	0.001*
*Yes*	937 (8.02)	242 (6.22)	302 (7.76)	393 (10.1)	
Heart problems (n, %)					
*No*	9936 (85.1)	3433 (88.2)	3346 (85.9)	3157 (81.1)	0.001*
*Yes*	1743 (14.9)	460 (11.8)	547 (14.1)	736 (18.9)	
Stroke (n, %)					
*No*	11390 (97.5)	3825 (98.3)	3807 (97.8)	3758 (96.5)	0.001*
*Yes*	289 (2.47)	68 (1.75)	86 (2.21)	135 (3.47)	
Dyslipidemia (n, %)					
*No*	10029 (85.9)	3503 (90.0)	3401 (87.4)	3125 (80.3)	0.001*
*Yes*	1650 (14.1)	390 (10.0)	492 (12.6)	768 (19.7)	
Drink (n, %)					
*No*	6266 (53.7)	2231 (57.3)	2035 (52.3)	2000 (51.4)	0.001*
*Yes*	5413 (46.3)	1662 (42.7)	1858 (47.7)	1893 (48.6)	
Smoke (n, %)					
*No*	6305 (54.0)	2214 (56.9)	2087 (53.6)	2004 (51.5)	0.001*
*Yes*	5374 (46.0)	1679 (43.1)	1806 (46.4)	1889 (48.5)	
SBP (mmHg, median (IQR))	125 (114, 140)	124 (112, 139)	126 (114, 140)	126 (113, 140)	0.336
DBP (mmHg, median(IQR))	74.0 (66.5, 82.5)	74.0 (66.5, 82.5)	74.0 (67.0, 82.5)	74.0 (67.0, 82.5)	0.470
BMI (median (IQR))	23.5 (21.2, 26.0)	23.3 (21.0, 25.9)	23.6 (21.3, 26.1)	23.5 (21.2, 26.1)	0.003*
Life event (n, %)					
*No*	10 185 (87.2)	3497 (89.8)	3412 (87.6)	3276 (84.2)	0.001*
*Yes*	1494 (12.8)	396 (10.2)	481 (12.4)	617 (15.8)	
Child death (n, %)					
*No*	10 516 (90.0)	3642 (93.6)	3515 (90.3)	3359 (86.3)	0.001*
*Yes*	1163 (9.96)	251 (6.45)	378 (9.71)	534 (13.7)	
Cognitive function (median (IQR))	16 (13, 19)	16 (13, 19)	16 (13, 19)	15 (12, 18)	0.001*
*Attention*	4 (3, 5)	4 (3, 5)	4 (3, 5)	4 (3, 5)	0.017*
*Orientation*	4 (3, 4)	4 (3, 4)	4 (3, 4)	4 (3, 4)	0.110
*Visuo-construction*	1 (1, 1)	1 (1, 1)	1 (0, 1)	1 (0, 1)	0.001*
*Episodic memory*	8 (5, 10)	8 (6, 10)	8 (6, 10)	7 (5, 10)	0.001*
PM_1_ (μg/m^3^, median (IQR))	31.1 (23.7, 39.7)	31.8 (25.3, 40.0)	33.9 (25.3, 43.3)	26.5 (20.9, 34.5)	0.001*
PM_2.5_ (μg/m^3^, median (IQR))	54.3 (41.3, 72.0)	59.4 (43.2, 71.4)	60.4 (43.8, 78.2)	48.5 (36.5, 59.1)	0.001*
PM_10_ (μg/m^3^, median (IQR))	93.9 (65.8, 120)	102 (70.0, 122)	106 (72.3, 134)	81.3 (58.7, 103)	0.001*
NO_2_ (μg/m^3^, median (IQR))	28.6 (22.0, 37.3)	29.2 (22.1, 38.0)	30.2 (23.7, 40.1)	26.5 (20.2, 33.8)	0.001*
O_3_ (μg/m^3^, median (IQR))	84.3 (81.0, 88.7)	84.5 (81.5, 88.9)	84.8 (82.5, 87.8)	83.5 (78.1, 90.0)	0.001*

BMI – body mass index, DBP – diastolic blood pressure, IQR – interquartile range, mmHg – milimetres of mercury, μg – micrometre, SBP – systolic blood pressure

*Statistically significant results (*P* < 0.05).

### Covariates selection

The results of uni root test suggested that the panel data are stationary while Hausman test indicated that fixed effect models were applicable to our analysis. Several significant associations were found between potential risk factors and cognitive function scores (**Table 2**): the older individuals (Coef. = –0.432, *P* = 0.003), who were married or partnered (Coef. = –0.668, *P* = 0.023), previously diagnosed with hypertension (Coef. = –0.669, *P* = 0.001) or stroke (Coef. = –2.388, *P* = 0.001), smoke (Coef. = –0.833, *P* = 0.001), experienced severe injuries (Coef. = –0.681, *P* = 0.006), loss a child previously (Coef. = –0.731, *P* = 0.001) were related to lower cognitive function scores, while those participate in social activities (Coef. = 0.366, *P* = 0.001) were more likely to get higher scores. After these eight covariates were jointly entered into the multifactor panel regression, age (Coef. = 0.274, *P* = 0.058) and marital status was excluded (Coef. = 0.544, *P* = 0.063) (**Table 3**).

**Table 2:** Univariate panel data linear regression results for the potential risk factors

Variables	Coef.	SE	t	*P-*value	95% CI
Age	−0.432	0.143	−3.02	0.003*	(−0.712, −0.152)
Marital status	−0.668	0.293	−2.28	0.023*	(−1.242, −0.093)
Hypertension	−0.669	0.198	−3.38	0.001*	(−1.057, −0.281)
Diabetes	−0.48	0.3	−1.6	0.109	(−1.068, 0.107)
Heart problems	−0.399	0.222	−1.8	0.072	(−0.833, 0.036)
Stroke	−2.388	0.449	−5.32	0.001*	(−3.269, −1.508)
Dyslipidemia	−0.063	0.183	−0.34	0.731	(−0.421, 0.295)
Drink	0.131	0.114	1.15	0.249	(−0.092, 0.355)
Smoke	−0.833	0.254	−3.28	0.001*	(−1.331, −0.335)
Social work	0.366	0.082	4.45	0.001*	(0.205, 0.205)
SBP	−0.001	0.003	−0.38	0.702	(−0.006, 0.004)
DBP	0.003	0.004	0.76	0.445	(−0.005, 0.011)
BMI	0.033	0.026	1.26	0.207	(−0.0182, 0.084)
Life event	−0.681	0.248	−2.75	0.006*	(−1.166, −0.196)
Child death	−0.731	0.193	−3.78	0.001*	(−1.110, −0.352)
Gender	omitted				
Education level	omitted				
Residence	omitted				

BMI – body mass index, CI – confidence interval, Coef. – coefficient, DBP – diastolic blood pressure, SE – standard error, SBP – systolic blood pressure

*Statistically significant results(*P* < 0.05).

**Table 3:** Multivariate panel data linear regression results for the potential risk factors

Variables	Coef.	SE	t	*P-*value	95% CI
Age	0.2737369	0.1444179	1.9	0.058	(−0.557, 0.009)
Marital status	0.5438455	0.2921423	1.86	0.063	(−1.117, 0.029)
Hypertension	0.4445062	0.1994057	2.23	0.026*	(−0.835, −0.054)
Stroke	2.2216450	0.4494784	4.94	0.001*	(−3.103, −1.341)
Smoke	0.6940425	0.2542378	2.73	0.006*	(−1.192, −0.196)
Social work	0.3772334	0.0820679	4.6	0.001*	(0.216, 0.538)
Life event	0.4888606	0.2483864	1.97	0.049*	(−0.976, −0.002)
Child death	0.6003717	0.1939802	3.1	0.002*	(−0.981, −0.220)

CI – confidence interval, Coef. – coefficient, SE – standard error

*Statistically significant results (*P* < 0.05).

### Effects of air pollutants on cognitive function

According to the results in models exploring the effect of single air pollutant (**Table 4**), PM_1_ (Coef. = −0.093, *P* = 0.001), PM_2.5_ (Coef. = −0.051, *P* = 0.001), PM_10_ (Coef. = −0.030, *P* = 0.001) and NO_2_ (Coef. = −0.094, *P* = 0.006) were negatively associated with cognitive function scores, suggesting that higher pollutant concentrations are associated with a greater likelihood of lower cognitive function test scores. No association between O_3_ (Coef. = −0.039, *P* = 0.117) and cognitive function has been observed. When the pollutants interaction (PM_1_, PM_2.5_, PM_10_, NO_2_ and O_3_) was added into the regression model with five pollutants, the results showed a significant negative association between pollutants interaction and cognitive function (Coef. = −2.89, *P* = 0.004) (**Table 5**).

**Table 4:** Panel data linear regression results for the effect of single air pollutant

Variables	PM_1_				PM_2.5_				PM_10_					NO_2_					O_3_			
	Coef.	*P-*value	95% CI		Coef.	*P-*value	95% CI		Coef.	*P-*value	95% CI		Coef.		*P-*value	95% CI		Coef.		*P-*value	95% CI	
			Lower	Upper			Lower	Upper			Lower	Upper				Lower	Upper				Lower	Upper
**Cognitive functioning**	**−0.093**	0.001*	**−0.136**	**−0.050**	**−0.051**	0.001*	**−0.073**	**−0.029**	**−0.030**	0.001*	**−0.043**	**−0.017**	**−0.094**		0.006*	**−0.161**	**−0.027**	**−0.039**		**0.117**	**−0.087**	**0.010**
**Attention**	**−0.014**	**0.072**	**−0.028**	**0.001**	**−0.007**	**0.090**	**−0.014**	**0.001**	**−0.003**	**0.192**	**−0.007**	**0.001**	**0.024**		0.038*	**0.001**	**0.047**	**−0.005**		**0.523**	**−0.022**	**0.011**
**Orientation**	**−0.011**	**0.065**	**−0.023**	**0.001**	**−0.006**	0.043*	**−0.012**	**0.000**	**−0.004**	0.015*	**−0.008**	**−0.001**	**−0.004**		**0.668**	**−0.022**	**0.014**	**−0.006**		**0.353**	**−0.019**	**0.007**
**Visuo-construction**	**−0.010**	0.001*	**−0.016**	**−0.005**	**−0.005**	0.001*	**−0.008**	**−0.002**	**−0.003**	0.001*	**−0.005**	**−0.001**	**−0.014**		0.002*	**−0.022**	**−0.005**	**−0.005**		**0.145**	**−0.011**	**0.002**
**Episodic memory**	**−0.059**	0.002*	**−0.095**	**−0.022**	**−0.033**	0.001*	**−0.052**	**−0.014**	**−0.019**	0.001*	**−0.030**	**−0.008**	**−0.101**		0.001*	**−0.158**	**−0.044**	**−0.022**		**0.289**	**−0.064**	**0.019**


CI – confidence interval, PM – particulate mater, NO_2_ – nitrogen dioxide, O_3_ – ozone

*Statistically significant results (*P* < 0.05). Adjusted for hypertension, stroke, smoke, social work, life event and child death in each model.

**Table 5:** Panel data linear regression results for the interaction of air pollutants

Variables	Coef.	SE	t	*P*-value	95% CI	
					**Lower**	**Upper**
Cognitive functioning	−2.82	0.966	−2.92	0.004*	−4.715	−0.926
Attention	0.393	0.333	1.18	0.239	−0.261	1.046
Orientation	−0.373	0.264	−1.41	0.159	−0.891	0.146
Visuo-construction	−0.12	0.125	−0.97	0.334	−0.365	0.124
Episodic memory	−2.72	0.824	−3.3	0.001*	−4.335	−1.104

CI – confidence interval, Coef. – coefficient, SE – standard error

*Statistically significant results (*P* < 0.05). Adjusted for hypertension, stroke, smoke, social work, life event and child death in each model. Total = lg(PM_1_*PM_2.5_*PM_10_*NO_2_*O_3_)

Additionally, when analysing the four sections of cognitive function (**Table 4**), PM_1_ (Coef. = −0.010, *P* = 0.001), PM_2.5_ (Coef. = −0.005, *P* = 0.001), PM_10_ (Coef. = −0.003, *P* = 0.001), and NO_2_ (Coef. = −0.014, *P* = 0.002) exhibited a negative correlation with visual construction; PM_1_ (Coef. = −0.059, *P* = 0.002), PM_2.5_ (Coef. = −0.033, *P* = 0.001), PM_10_ (Coef. = −0.019, *P* = 0.001), and NO_2_ (Coef. = −0.101, *P* = 0.001) displayed negative effects on episodic memory. Both PM_10_ (Coef. = −0.004, *P* = 0.015) and O_3_ (Coef. = −0.006, *P* = 0.019) were found to be negatively associated with orientation. However, NO_2_ (Coef. = −0.038, *P* = 0.001) showed a positive influence on attention. Furthermore, the interaction of the five air pollutants demonstrated a negative impact specifically on episodic memory (Coef. = −2.72, *P* = 0.001) (**Table 5**). The comprehensive overview of the results from each model can be found in Tables S2–S31 in the **Online Supplementary Document**. The results of repeated modeling using White's heteroskedasticity-robust standard errors consistent with the above that using default standard errors (Tables S32–S33 in the **Online Supplementary Document**).

## DISCUSSION

In this panel study encompassing 3893 individuals aged 45 years and older in China, the air pollutants concentrations in the study area peaked in 2013, followed by a subsequent reduction in 2015. Our investigation delved into the potential association between exposure to air pollutants and cognitive function. Our results suggest that increased exposure to air pollutants may be associated with poorer cognitive function. Specifically, we found a negative association between air pollutant interaction and cognitive function, adjusting for several factors such as smoking, life events, and other potential confounders. Interestingly, we also noted that each single air pollutant exposure was significantly associated with cognitive function, with the exception of O_3_. This exception may be attributed to the necessity for longer observation periods to fully capture the cognitive function changes resulting from O_3_ exposure.

Similarly, the research based on China Health and Retirement Longitudinal Study (CLHLS) reported that increased exposure to PM_2.5_ affects cognitive decline among older adults. In other words, a negative correlation was found among participants aged 65 and above between the change in Mini-Mental State Examination (MMSE) scores over four years and their exposure to PM_2.5_, while a positive correlation was observed with O_3_ [31]. Our finding also aligns with studies conducted in Chinese male military veterans, revealing that high levels of PM_1_ and NO_2_ concentration increase the risk of cognitive decline [32]. A nationwide large-scale prospective cohort study conducted in Chinese middle-aged and older adults also demonstrated that long-term exposure to PM_2.5_ hastens cognitive decline, impacting mathematical skills (coefficients of −0.159 and −0.244 for total cognitive and mathematical scores, respectively) [33]. Research from various parts of the world has also shown consistent results with our findings. For instance, a cohort study from the USA reveals that older women living in areas where air quality improved over the past decade exhibited cognitive function equivalent to being 0.9–1.2 years younger, particularly in terms of episodic memory, which was comparable to being 1.4–1.6 years younger [34]. Moreover, long-term exposure to high levels of PM_2.5_ and PM_2.5-10_ was associated with a decline of 0.18 and 0.2 points in cognitive function scores, respectively [35].

Further, the epidemiological findings are supported by related mechanistic studies. Existing research indicates that particulate matter can penetrate the blood-lung and blood-brain barriers, exerting oxidative effects directly or indirectly and altering lipids, proteins, and DNA, which can particularly impact older adults [36]. On the other hand, as suggested by Salvi et al., NO_2_ may increase oxidative stress levels and generate free radicals in the prefrontal cortex and hippocampus, both of which play a role in regulating cognitive function [37]. Recent domestic and international studies also failed to find a link between O_3_ and cognitive function [38]. This might be attributed to the role that O_3_ acts as a protective factor against low-grade systemic inflammation, however, this hypothesis still requires conclusive evidence.

Regarding specific cognitive performance, our research found negative effects of PM_1_, PM_2.5_, PM_10_, and NO_2_ on visual-construction and episodic memory, while PM_10_ and O_3_ showed negative associations with orientation functioning. Consistent with our result, existing evidence found that for every 1 μg/m^3^ increase in PM_2.5_, memory scores decreased by 0.274 (95% CI = −0.361, −0.188) based on the China Family Panel Studies (CFPS). It is possibly caused by the association between PM_2.5_ and the progression of gray matter atrophy in brain regions susceptible to Alzheimer disease and episodic memory, according to the findings from the Women's Health Initiative Memory Study (WHIMS). In the results of a two-dimensional cognitive function test that composed of working memory and orientation, Ailshire et al. found a 1.5 times higher error rate among American adults aged 55 and older living in areas with high PM_2.5_ concentrations than those with low concentrations [39]. A study based on National Health and Nutrition Examination Surveys (NHANES) III showed that higher PM_10_ or O_3_ exposure is associated with poorer performance on memory and visual-construction [21]. Data from the Taiwan Biobank indicated that NO_2_ exposure were inversely associated with the performance in visual construction. However, in this study, NO_2_ demonstrated a possible positive impact on the attention domain, specifically in tasks involving calculation ability. The result suggests the subtle negative effects of NO_2_ on cognitive function, are likely influenced by confounding factors. We speculate that this effect might be attributed to the interaction and conversion between NO and NO_2_. Given that NO is closely associated with synaptic transmission, this interaction could potentially influence cognitive function. Furthermore, this finding emphasises the importance of conducting longer-term longitudinal studies to comprehensively explore the relationship between nitrogen dioxide exposure and cognitive function.

Therefore, we emphasise the impact of potential confounding factors and attach importance to corresponding improvements. In our study, hypertension, stroke, smoking, and social activities showed a significant impact on cognitive function. Previous research has also shown that long-term exposure to air pollution is associated with a wide range of health outcomes, including chronic diseases such as hypertension and stroke [40]. However, it is worth noting that smoking exhibited a positive effect (Coef. = 0.137) when investigating the impact of PM_2.5_ on orientation. It may due to the short term effect of nicotine on the cholinergic system, which may lead to neuroprotective effects and positive effects on certain cognitive domains under certain conditions [41]. These findings indicate the need for targeted interventions and support for individuals affected by poor living circumstances or health habits.

This study has two major strengths. On one hand, panel data regression were introduced to the analysis, allowing for the consideration of time-invariant characteristics and capturing individual-specific dynamics over time. On the other hand, the lag effects of air pollutants on cognitive functioning were incorporated, ensuring a clear temporal sequence in the exploration of causality between them. Nevertheless, several limitations of our study warrant consideration. First, the observation period was not long enough to gather monitoring data for causal inferences. Cohort studies or long-term observational researches are needed for implementation. Second, not all potential confounding covariates were included in our study. Potential confounders, such as financial burden [42], emotional relationships [43], noise, neighborhood relationships, sleep time, green space in residential areas, indoor air pollution caused by solid fuel usage, etc. may introduce biases in effect estimation [44]. This emphasises the need for improved methods to measure the exposure of different individuals residing in the same location accurately. Third, the cognitive function assessment used in this study relied on questionnaire surveys rather than rigorous diagnostic testing, which may present challenges in regions where dialects are prevalent. Fourthly, to ensure the integrity of the panel data, individuals with missing records were excluded, which could potentially affect the representativeness of the participants. In addition, there is a need for a more precise approach to assess the individual-level pollutant exposure among participants. For example, utilising geographic information system in combination with data from meteorological observation station to determine air quality in different areas within cities. Furthermore, the environmental exposure in this study did not account for the spatial heterogeneity of air pollutants and did not include factors such as climate and temperature, which may not be able to capture the precise exposure and reduce the accuracy of results [29].

It is well-known that there is a time window spanning several decades from the onset of neuropathological changes to clinical symptoms of cognitive decline. Therefore, it provides a broad opportunity to prevent or delay cognitive impairment. In China, where aging is a pressing public health issue and the burden of dementia is substantial, it is essential to implement control and preventive measures during the preclinical phase. Though the rapid urbanisation process has posed numerous challenges to the implementation of clean air policies. However, implementing environmental protection policies to decrease the burden is undeniably cost-effective. Existing evidence shows that the implementation of the 1990 Clean Air Act Amendments (CAAA) in the USA led to a decrease of approximately 15 000 hospitalisations due to dementia annually [45].

From 2011 to 2013, China experienced rapid industrialisation and urbanisation, leading to a worsening of air pollution issues [46]. Since the release of the ‘China’s Clean Air Act’ in 2013, environmental regulations have been strengthened. China has implemented a lot of measures such as eliminating backward productivity, curbing the disorderly expansion of industrial scale, and promoting energy conservation and emission reduction. These efforts are aimed at promoting the optimisation of the industrial structure, achieving coordinated control of multiple pollutants, and improving air quality. The results of our study demonstrated the decrease of air pollution in Chinese provinces from 2013 to 2015, which suggests the effectiveness of China's clean air action. Also, through literature review, we recognised a trend in China where economic development and urbanisation process are gradually increasing from west to east and from north to south. This trend is accompanied by higher levels of education and urban residential rate, which correlate with a lower proportion of cognitive decline. In this circumstance, the negative correlation between pollution and cognitive functioning indicates that air pollution control plays a protective role in maintaining the cognitive function among middle-aged and elderly population. Thus, it is crucial to pursue sustainable economic growth. On one hand, enhancing economic strength enables the government to provide better health care and hygiene conditions for the public. On the other hand, promoting the development of innovative clean energy technologies and providing incentives for new energy industry development can effectively reduce atmospheric pollutant emissions from the source. In addition, the results of this study underscore the necessity of long-term monitoring of major air pollutants. For residents at middle-aged or older, reducing nicotine intake, going out for social activities, and controlling the cardiovascular and cerebrovascular diseases progression would contribute to slowing down the pace of cognitive decline. This highlights the importance of increasing input in health education for middle-aged and elderly population, include promoting the use of air purifiers and N95 respirator masks to reduce indoor and outdoor exposure to air pollutants, which may help prevent cognitive impairment among vulnerable population and reduce national health care costs.

In terms of future research directions, it is imperative to use more accurate tools for cognitive function measurement. Additionally, there is a need for a more precise approach to assess the individual-level pollutant exposure among participants. For example, utilising Geographic Information System in combination with data from meteorological observation station to determine air quality in different areas within cities. These efforts are crucial for advancing our understanding and effectively addressing the health challenges posed by air pollution.

## CONCLUSIONS

Our results reveal that major air pollutants exposure brings adverse effects to cognitive function among middle-aged and elderly Chinese. The findings support the need for stricter environmental regulations and policies to mitigate air pollution and protect cognitive health.

## Additional material


Online Supplementary Document
