# Supplementary material for: Effects of major air pollutants on cognitive function in middle-aged and elderly adults: Panel data evidence from China Health and Retirement Longitudinal Study
Source: J Glob Health. 2024 Nov 8;14:04153. doi: 10.7189/jogh.14.04153 (PMC11544526; doi:10.7189/jogh.14.04153)
Supplement: Online Supplementary Document [file jogh-14-04153-s001.pdf]

Supplementary

Table S1 Linear trend test results

**Pollutants**	**F**	***P***
PM_1	383.756	0.001
PM_{25}	400.944	0.001
PM_{10}	353.816	0.001
NO_2	155.91	0.001
O_3	25.918	0.001

*Statistically significant results are bolded in the table ($P < 0.05$)

Table S2 Panel data linear regression results for the effect of PM_1 on cognitive function

**Variables**	**Coef.**	**Std.Err**	**t**	***P***	**95% CI**	
					**Lower**	**Upper**
PM_1	-0.093	0.022	-4.260	**0.001**	-0.136	-0.050
Hypertension	-0.596	0.282	-2.120	**0.034**	-1.149	-0.044
Stroke	-2.514	0.586	-4.290	**0.001**	-3.663	-1.365
Smoke	-1.081	0.452	-2.390	**0.017**	-1.968	-0.195
Social work	0.319	0.116	2.740	**0.006**	0.091	0.547
Life event	-0.160	0.354	-0.450	0.651	-0.853	0.533
Child Death	-0.471	0.300	-1.570	0.117	-1.059	0.118

*Statistically significant results are bolded in the table ($P < 0.05$). Adjusted for hypertension, stroke, smoke, social work, life event and child death in each model.

Table S3 Panel data linear regression results for the effect of $PM_{2.5}$ on cognitive function

**Variables**	**Coef.**	**Std.Err**	**t**	***P***	**95% CI**	
					**Lower**	**Upper**
$PM_{2.5}$	-0.051	0.011	-4.490	**0.001**	-0.073	-0.029
Hypertension	-0.580	0.282	-2.060	**0.040**	-1.133	-0.028
Stroke	-2.501	0.586	-4.270	**0.001**	-3.650	-1.352
Smoke	-1.062	0.452	-2.350	**0.019**	-1.949	-0.175
Social work	0.317	0.116	2.730	**0.006**	0.089	0.545
Life event	-0.139	0.354	-0.390	0.695	-0.833	0.555
Child Death	-0.461	0.300	-1.540	0.125	-1.049	0.127

*Statistically significant results are bolded in the table ($P < 0.05$). Adjusted for hypertension, stroke, smoke, social work, life event and child death in each model.

Table S4 Panel data linear regression results for the effect of PM_{10} on cognitive function

Variables	Coef.	Std.Err	t	*P*	95% CI	
					Lower	Upper
PM_{10}	-0.030	0.007	-4.500	**0.001**	-0.043	-0.017
Hypertension	-0.574	0.282	-2.030	**0.042**	-1.127	-0.021
Stroke	-2.491	0.586	-4.250	**0.001**	-3.641	-1.342
Smoke	-1.055	0.452	-2.330	**0.020**	-1.942	-0.168
Social work	0.318	0.116	2.740	**0.006**	0.090	0.546
Life event	-0.125	0.354	-0.350	0.724	-0.819	0.569
Child Death	-0.460	0.300	-1.530	0.126	-1.048	0.129

*Statistically significant results are bolded in the table ($P < 0.05$). Adjusted for hypertension, stroke, smoke, social work, life event and child death in each model.

Table S5 Panel data linear regression results for the effect of NO_2 on cognitive function

Variables	Coef.	Std.Err	t	*P*	95% CI	
					Lower	Upper
NO_2	-0.094	0.034	-2.750	**0.006**	-0.161	-0.027
Hypertension	-0.695	0.281	-2.480	**0.013**	-1.245	-0.145
Stroke	-2.588	0.586	-4.410	**0.001**	-3.738	-1.438
Smoke	-1.194	0.452	-2.640	**0.008**	-2.079	-0.308
Social work	0.331	0.117	2.840	**0.005**	0.102	0.559
Life event	-0.247	0.353	-0.700	0.484	-0.939	0.445
Child Death	-0.529	0.300	-1.760	0.078	-1.117	0.059

*Statistically significant results are bolded in the table ($P < 0.05$). Adjusted for hypertension, stroke, smoke, social work, life event and child death in each model.

Table S6 Panel data linear regression results for the effect of O_3 on cognitive function

Variables	Coef.	Std.Err	t	*P*	95% CI	
					Lower	Upper
O_3	-0.039	0.025	-1.570	0.117	-0.087	0.010
Hypertension	-0.753	0.280	-2.690	**0.007**	-1.301	-0.204
Stroke	-2.651	0.586	-4.520	**0.001**	-3.801	-1.502
Smoke	-1.280	0.450	-2.840	**0.005**	-2.163	-0.397
Social work	0.348	0.116	2.990	**0.003**	0.120	0.576
Life event	-0.320	0.352	-0.910	0.364	-1.010	0.371
Child Death	-0.579	0.300	-1.930	0.053	-1.166	0.008

*Statistically significant results are bolded in the table ($P < 0.05$). Adjusted for hypertension, stroke, smoke, social work, life event and child death in each model.

Table S7 Panel data linear regression results for the effect of PM_1 on attention

Variables	Coef.	Std.Err	t	*P*	95% CI	
					Lower	Upper
PM_1	-0.014	0.008	-1.800	0.072	-0.028	0.001
Hypertension	-0.003	0.097	-0.030	0.972	-0.194	0.187
Stroke	-0.736	0.202	-3.640	**0.001**	-1.132	-0.340
Smoke	-0.321	0.156	-2.060	**0.039**	-0.627	-0.016
Social work	0.092	0.040	2.290	**0.022**	0.013	0.170
Life event	-0.019	0.122	-0.160	0.876	-0.258	0.220
Child Death	-0.054	0.104	-0.520	0.602	-0.257	0.149

*Statistically significant results are bolded in the table ($P < 0.05$). Adjusted for hypertension, stroke, smoke, social work, life event and child death in each model.

Table S8 Panel data linear regression results for the effect of $PM_{2.5}$ on attention

Variables	Coef.	Std.Err	t	*P*	95% CI	
					Lower	Upper
$PM_{2.5}$	-0.007	0.004	-1.700	0.090	-0.014	0.001
Hypertension	-0.004	0.097	-0.040	0.969	-0.195	0.187
Stroke	-0.737	0.202	-3.640	**0.001**	-1.133	-0.340
Smoke	-0.322	0.156	-2.070	**0.039**	-0.628	-0.016
Social work	0.092	0.040	2.290	**0.022**	0.013	0.171
Life event	-0.019	0.122	-0.150	0.878	-0.258	0.221
Child Death	-0.055	0.104	-0.530	0.598	-0.258	0.148

*Statistically significant results are bolded in the table ($P < 0.05$). Adjusted for hypertension, stroke, smoke, social work, life event and child death in each model.

Table S9 Panel data linear regression results for the effect of PM_{10} on attention

Variables	Coef.	Std.Err	t	*P*	95% CI	
					Lower	Upper
PM_{10}	-0.003	0.002	-1.310	0.192	-0.007	0.001
Hypertension	-0.009	0.097	-0.090	0.928	-0.200	0.182
Stroke	-0.740	0.202	-3.660	**0.001**	-1.137	-0.344
Smoke	-0.329	0.156	-2.110	**0.035**	-0.635	-0.023
Social work	0.093	0.040	2.320	**0.020**	0.015	0.172
Life event	-0.023	0.122	-0.190	0.853	-0.262	0.217
Child Death	-0.058	0.104	-0.560	0.573	-0.261	0.145

*Statistically significant results are bolded in the table ($P < 0.05$). Adjusted for hypertension, stroke, smoke, social work, life event and child death in each model.

Table S10 Panel data linear regression results for the effect of NO_2 on attention

Variables	Coef.	Std.Err	t	*P*	95% CI	
					Lower	Upper
NO_2	0.024	0.012	2.080	**0.038**	0.001	0.047
Hypertension	-0.046	0.097	-0.480	0.631	-0.236	0.143
Stroke	-0.777	0.202	-3.850	**0.001**	-1.173	-0.381
Smoke	-0.381	0.156	-2.450	**0.014**	-0.686	-0.076
Social work	0.103	0.040	2.570	**0.010**	0.025	0.182
Life event	-0.061	0.122	-0.500	0.618	-0.299	0.178
Child Death	-0.087	0.103	-0.840	0.399	-0.290	0.115

*Statistically significant results are bolded in the table ($P < 0.05$). Adjusted for hypertension, stroke, smoke, social work, life event and child death in each model.

Table S11 Panel data linear regression results for the effect of O_3 on attention

Variables	Coef.	Std.Err	t	*P*	95% CI	
					Lower	Upper
O_3	-0.005	0.009	-0.640	0.523	-0.022	0.011
Hypertension	-0.026	0.096	-0.270	0.786	-0.215	0.163
Stroke	-0.756	0.202	-3.750	**0.001**	-1.152	-0.360
Smoke	-0.350	0.155	-2.260	**0.024**	-0.654	-0.046
Social work	0.096	0.040	2.400	**0.017**	0.017	0.175
Life event	-0.042	0.121	-0.350	0.728	-0.280	0.196
Child Death	-0.070	0.103	-0.680	0.498	-0.272	0.132

*Statistically significant results are bolded in the table ($P < 0.05$). Adjusted for hypertension, stroke, smoke, social work, life event and child death in each model.

Table S12 Panel data linear regression results for the effect of PM_1 on orientation

Variables	Coef.	Std.Err	t	*P*	95% CI	
					Lower	Upper
PM_1	-0.011	0.006	-1.850	0.065	-0.023	0.001
Hypertension	0.083	0.077	1.080	0.280	-0.068	0.234
Stroke	-0.096	0.160	-0.600	0.551	-0.410	0.218
Smoke	0.134	0.124	1.080	0.278	-0.108	0.376
Social work	0.016	0.032	0.490	0.624	-0.047	0.078
Life event	0.068	0.097	0.700	0.483	-0.122	0.257
Child Death	-0.025	0.082	-0.300	0.762	-0.186	0.136

*Statistically significant results are bolded in the table ($P < 0.05$). Adjusted for hypertension, stroke, smoke, social work, life event and child death in each model.

Table S13 Panel data linear regression results for the effect of $PM_{2.5}$ on orientation

**Variables**	**Coef.**	**Std.Err**	**t**	***P***	**95% CI**	
					**Lower**	**Upper**
$PM_{2.5}$	-0.006	0.003	-2.020	**0.043**	-0.012	0.000
Hypertension	0.086	0.077	1.120	0.265	-0.065	0.237
Stroke	-0.093	0.160	-0.580	0.560	-0.407	0.221
Smoke	0.137	0.124	1.110	0.266	-0.105	0.380
Social work	0.015	0.032	0.480	0.633	-0.047	0.078
Life event	0.071	0.097	0.740	0.462	-0.118	0.261
Child Death	-0.023	0.082	-0.280	0.778	-0.184	0.138

*Statistically significant results are bolded in the table ($P < 0.05$). Adjusted for hypertension, stroke, smoke, social work, life event and child death in each model.

Table S14 Panel data linear regression results for the effect of PM_{10} on orientation

**Variables**	**Coef.**	**Std.Err**	**t**	***P***	**95% CI**	
					**Lower**	**Upper**
PM_{10}	-0.004	0.002	-2.430	**0.015**	-0.008	-0.001
Hypertension	0.091	0.077	1.190	0.236	-0.060	0.243
Stroke	-0.088	0.160	-0.550	0.583	-0.402	0.226
Smoke	0.144	0.124	1.170	0.243	-0.098	0.387
Social work	0.014	0.032	0.450	0.650	-0.048	0.077
Life event	0.078	0.097	0.800	0.423	-0.112	0.267
Child Death	-0.020	0.082	-0.240	0.809	-0.181	0.141

*Statistically significant results are bolded in the table ($P < 0.05$). Adjusted for hypertension, stroke, smoke, social work, life event and child death in each model.

Table S15 Panel data linear regression results for the effect of NO_2 on orientation

**Variables**	**Coef.**	**Std.Err**	**t**	***P***	**95% CI**	
					**Lower**	**Upper**
NO_2	-0.004	0.009	-0.430	0.668	-0.022	0.014
Hypertension	0.066	0.077	0.860	0.388	-0.084	0.216
Stroke	-0.110	0.160	-0.690	0.492	-0.424	0.204
Smoke	0.113	0.123	0.910	0.361	-0.129	0.354
Social work	0.019	0.032	0.590	0.554	-0.044	0.081
Life event	0.052	0.096	0.540	0.589	-0.137	0.241
Child Death	-0.036	0.082	-0.440	0.657	-0.197	0.124

*Statistically significant results are bolded in the table ($P < 0.05$). Adjusted for hypertension, stroke, smoke, social work, life event and child death in each model.

Table S16 Panel data linear regression results for the effect of O_3 on orientation

Variables	Coef.	Std.Err	t	*P*	95% CI	
					**Lower**	**Upper**
O_3	-0.006	0.007	-0.930	0.353	-0.019	0.007
Hypertension	0.065	0.076	0.860	0.392	-0.084	0.215
Stroke	-0.111	0.160	-0.700	0.486	-0.425	0.202
Smoke	0.111	0.123	0.910	0.365	-0.130	0.352
Social work	0.019	0.032	0.590	0.556	-0.044	0.081
Life event	0.049	0.096	0.510	0.611	-0.140	0.237
Child Death	-0.037	0.082	-0.460	0.648	-0.198	0.123

*Statistically significant results are bolded in the table ($P < 0.05$). Adjusted for hypertension, stroke, smoke, social work, life event and child death in each model.

Table S17 Panel data linear regression results for the effect of PM_1 on visuo-construction

Variables	Coef.	Std.Err	t	*P*	95% CI	
					**Lower**	**Upper**
PM_1	-0.010	0.003	-3.610	**0.001**	-0.016	-0.005
Hypertension	-0.017	0.036	-0.470	0.642	-0.088	0.054
Stroke	-0.102	0.076	-1.350	0.178	-0.250	0.046
Smoke	-0.033	0.058	-0.570	0.567	-0.148	0.081
Social work	-0.004	0.015	-0.300	0.767	-0.034	0.025
Life event	0.037	0.046	0.820	0.413	-0.052	0.127
Child Death	0.020	0.039	0.520	0.602	-0.056	0.096

*Statistically significant results are bolded in the table ($P < 0.05$). Adjusted for hypertension, stroke, smoke, social work, life event and child death in each model.

Table S18 Panel data linear regression results for the effect of $PM_{2.5}$ on visuo-construction

Variables	Coef.	Std.Err	t	*P*	95% CI	
					**Lower**	**Upper**
$PM_{2.5}$	-0.005	0.001	-3.590	**0.001**	-0.008	-0.002
Hypertension	-0.016	0.036	-0.450	0.653	-0.088	0.055
Stroke	-0.102	0.076	-1.340	0.179	-0.250	0.047
Smoke	-0.033	0.058	-0.560	0.575	-0.147	0.082
Social work	-0.004	0.015	-0.290	0.769	-0.034	0.025
Life event	0.038	0.046	0.840	0.399	-0.051	0.128
Child Death	0.020	0.039	0.530	0.598	-0.055	0.096

*Statistically significant results are bolded in the table ($P < 0.05$). Adjusted for hypertension, stroke, smoke, social work, life event and child death in each model.

Table S19 Panel data linear regression results for the effect of PM_{10} on visuo-construction

Variables	Coef.	Std.Err	t	*P*	95% CI	
					Lower	Upper
PM_{10}	-0.003	0.001	-3.670	**0.001**	-0.005	-0.001
Hypertension	-0.015	0.036	-0.420	0.676	-0.087	0.056
Stroke	-0.100	0.076	-1.320	0.186	-0.248	0.048
Smoke	-0.031	0.058	-0.540	0.590	-0.146	0.083
Social work	-0.004	0.015	-0.290	0.770	-0.034	0.025
Life event	0.040	0.046	0.880	0.377	-0.049	0.130
Child Death	0.021	0.039	0.540	0.590	-0.055	0.097

*Statistically significant results are bolded in the table ($P < 0.05$). Adjusted for hypertension, stroke, smoke, social work, life event and child death in each model.

Table S20 Panel data linear regression results for the effect of NO_2 on visuo-construction

Variables	Coef.	Std.Err	t	*P*	95% CI	
					Lower	Upper
NO_2	-0.014	0.004	-3.090	**0.002**	-0.022	-0.005
Hypertension	-0.025	0.036	-0.700	0.487	-0.096	0.046
Stroke	-0.107	0.076	-1.420	0.156	-0.256	0.041
Smoke	-0.042	0.058	-0.720	0.472	-0.156	0.072
Social work	-0.004	0.015	-0.270	0.788	-0.033	0.025
Life event	0.030	0.046	0.670	0.505	-0.059	0.120
Child Death	0.016	0.039	0.410	0.680	-0.060	0.092

*Statistically significant results are bolded in the table ($P < 0.05$). Adjusted for hypertension, stroke, smoke, social work, life event and child death in each model.

Table S21 Panel data linear regression results for the effect of O_3 on visuo-construction

Variables	Coef.	Std.Err	t	*P*	95% CI	
					Lower	Upper
O_3	-0.005	0.003	-1.460	0.145	-0.011	0.002
Hypertension	-0.034	0.036	-0.940	0.348	-0.105	0.037
Stroke	-0.117	0.076	-1.550	0.122	-0.265	0.031
Smoke	-0.055	0.058	-0.950	0.345	-0.169	0.059
Social work	-0.001	0.015	-0.090	0.928	-0.031	0.028
Life event	0.020	0.045	0.440	0.663	-0.069	0.109
Child Death	0.008	0.039	0.220	0.827	-0.067	0.084

*Statistically significant results are bolded in the table ($P < 0.05$). Adjusted for hypertension, stroke, smoke, social work, life event and child death in each model.

Table S22 Panel data linear regression results for the effect of PM_1 on episodic memory

**Variables**	**Coef.**	**Std.Err**	**t**	***P***	**95% CI**	
					**Lower**	**Upper**
PM_1	-0.059	0.019	-3.130	**0.002**	-0.095	-0.022
Hypertension	-0.659	0.241	-2.740	**0.006**	-1.131	-0.188
Stroke	-1.580	0.500	-3.160	**0.002**	-2.561	-0.599
Smoke	-0.860	0.386	-2.230	**0.026**	-1.617	-0.104
Social work	0.216	0.099	2.180	**0.030**	0.021	0.411
Life event	-0.246	0.302	-0.810	0.415	-0.838	0.346
Child Death	-0.412	0.256	-1.610	0.108	-0.914	0.091

*Statistically significant results are bolded in the table ($P < 0.05$). Adjusted for hypertension, stroke, smoke, social work, life event and child death in each model.

Table S23 Panel data linear regression results for the effect of $PM_{2.5}$ on episodic memory

**Variables**	**Coef.**	**Std.Err**	**t**	***P***	**95% CI**	
					**Lower**	**Upper**
$PM_{2.5}$	-0.033	0.010	-3.390	**0.001**	-0.052	-0.014
Hypertension	-0.646	0.241	-2.680	**0.007**	-1.118	-0.174
Stroke	-1.570	0.500	-3.140	**0.002**	-2.551	-0.589
Smoke	-0.844	0.386	-2.190	**0.029**	-1.602	-0.087
Social work	0.214	0.099	2.160	**0.031**	0.020	0.409
Life event	-0.230	0.302	-0.760	0.447	-0.822	0.363
Child Death	-0.404	0.256	-1.580	0.115	-0.906	0.099

*Statistically significant results are bolded in the table ($P < 0.05$). Adjusted for hypertension, stroke, smoke, social work, life event and child death in each model.

Table S24 Panel data linear regression results for the effect of PM_{10} on episodic memory

**Variables**	**Coef.**	**Std.Err**	**t**	***P***	**95% CI**	
					**Lower**	**Upper**
PM_{10}	-0.019	0.006	-3.410	**0.001**	-0.030	-0.008
Hypertension	-0.641	0.241	-2.660	**0.008**	-1.114	-0.169
Stroke	-1.563	0.500	-3.120	**0.002**	-2.544	-0.582
Smoke	-0.839	0.386	-2.170	**0.030**	-1.597	-0.082
Social work	0.215	0.099	2.160	**0.031**	0.020	0.409
Life event	-0.220	0.302	-0.730	0.466	-0.813	0.373
Child Death	-0.402	0.256	-1.570	0.116	-0.905	0.100

*Statistically significant results are bolded in the table ($P < 0.05$). Adjusted for hypertension, stroke, smoke, social work, life event and child death in each model.

Table S25 Panel data linear regression results for the effect of NO_2 on episodic memory

**Variables**	**Coef.**	**Std.Err**	**t**	***P***	**95% CI**	
					**Lower**	**Upper**
NO_2	-0.101	0.029	-3.460	**0.001**	-0.158	-0.044
Hypertension	-0.689	0.239	-2.880	**0.004**	-1.158	-0.220
Stroke	-1.593	0.500	-3.190	**0.001**	-2.574	-0.613
Smoke	-0.883	0.385	-2.290	**0.022**	-1.638	-0.129
Social work	0.213	0.099	2.140	**0.032**	0.018	0.407
Life event	-0.269	0.301	-0.890	0.372	-0.859	0.321
Child Death	-0.422	0.256	-1.650	0.099	-0.923	0.080

*Statistically significant results are bolded in the table ($P < 0.05$). Adjusted for hypertension, stroke, smoke, social work, life event and child death in each model.

Table S26 Panel data linear regression results for the effect of O_3 on episodic memory

**Variables**	**Coef.**	**Std.Err**	**t**	***P***	**95% CI**	
					**Lower**	**Upper**
O_3	-0.022	0.021	-1.060	0.289	-0.064	0.019
Hypertension	-0.758	0.239	-3.180	**0.001**	-1.226	-0.290
Stroke	-1.667	0.500	-3.330	**0.001**	-2.647	-0.687
Smoke	-0.986	0.384	-2.570	**0.010**	-1.739	-0.233
Social work	0.235	0.099	2.360	**0.018**	0.040	0.429
Life event	-0.346	0.301	-1.150	0.250	-0.935	0.243
Child Death	-0.480	0.255	-1.880	0.060	-0.981	0.021

*Statistically significant results are bolded in the table ($P < 0.05$). Adjusted for hypertension, stroke, smoke, social work, life event and child death in each model.

Table S27 Panel data linear regression results for the effect of air pollutants' interaction on cognitive function

Variables	Coef.	Std.Err	t	*P*	95% Confidential interval	
					**Lower**	**Upper**
Total	-2.820	0.966	-2.920	**0.004**	-4.715	-0.926
PM_1	0.346	0.181	1.910	0.056	-0.009	0.702
$PM_{2.5}$	-0.152	0.114	-1.330	0.182	-0.375	0.071
PM_{10}	0.016	0.028	0.560	0.572	-0.039	0.070
NO_2	0.090	0.061	1.460	0.145	-0.031	0.210
O_3	0.015	0.032	0.480	0.628	-0.047	0.077
Hypertension	-0.542	0.282	-1.920	0.055	-1.096	0.011
Stroke	-2.477	0.586	-4.230	**0.000**	-3.626	-1.329
Smoke	-1.004	0.453	-2.220	**0.027**	-1.892	-0.117
Social work	0.292	0.116	2.510	**0.012**	0.064	0.520
Life event	-0.062	0.355	-0.170	0.862	-0.757	0.634
Child Death	-0.404	0.300	-1.340	0.179	-0.992	0.185

*Statistically significant results are bolded in the table ($P < 0.05$). Adjusted for hypertension, stroke, smoke, social work, life event and child death in each model.

Table S28 Panel data linear regression results for the effect of air pollutants' interaction on attention

Variables	Coef.	Std.Err	t	*P*	95% Confidential interval	
					**Lower**	**Upper**
Total	0.393	0.333	1.180	0.239	-0.261	1.046
PM_1	-0.052	0.063	-0.830	0.405	-0.175	0.071
$PM_{2.5}$	-0.007	0.039	-0.190	0.850	-0.084	0.070
PM_{10}	0.007	0.010	0.720	0.473	-0.012	0.026
NO_2	0.015	0.021	0.720	0.472	-0.026	0.057
O_3	-0.013	0.011	-1.240	0.216	-0.035	0.008
Hypertension	-0.024	0.097	-0.240	0.807	-0.215	0.167
Stroke	-0.758	0.202	-3.750	**0.000**	-1.154	-0.361
Smoke	-0.352	0.156	-2.250	**0.024**	-0.658	-0.046
Social work	0.099	0.040	2.470	**0.013**	0.021	0.178
Life event	-0.051	0.122	-0.420	0.677	-0.291	0.189
Child Death	-0.075	0.104	-0.720	0.472	-0.278	0.129

*Statistically significant results are bolded in the table ($P < 0.05$). Adjusted for hypertension, stroke, smoke, social work, life event and child death in each model. A phase lag has been set at the effect of air pollutants, a phase equals to two years. Total=$lg(PM_1*PM_{2.5}*PM_{10}*NO_2*O_3)$

Table S29 Panel data linear regression results for the effect of air pollutants' interaction on orientation

Variables	Coef.	Std.Err	t	*P*	95% Confidential interval	
					Lower	Upper
Total	-0.373	0.264	-1.410	0.159	-0.891	0.146
PM_1	0.053	0.050	1.070	0.284	-0.044	0.150
$PM_{2.5}$	-0.005	0.031	-0.150	0.883	-0.066	0.056
PM_{10}	-0.010	0.008	-1.310	0.190	-0.025	0.005
NO_2	0.023	0.017	1.390	0.164	-0.010	0.056
O_3	0.002	0.009	0.270	0.790	-0.015	0.019
Hypertension	0.092	0.077	1.190	0.233	-0.059	0.244
Stroke	-0.089	0.160	-0.560	0.578	-0.403	0.225
Smoke	0.144	0.124	1.160	0.245	-0.099	0.387
Social work	0.014	0.032	0.440	0.662	-0.049	0.076
Life event	0.086	0.097	0.880	0.377	-0.105	0.276
Child Death	-0.018	0.082	-0.210	0.830	-0.179	0.143

*Statistically significant results are bolded in the table ($P < 0.05$). Adjusted for hypertension, stroke, smoke, social work, life event and child death in each model. A phase lag has been set at the effect of air pollutants, a phase equals to two years. Total=lg(PM_1*$PM_{2.5}$*PM_{10}*NO_2*O_3)

Table S30 Panel data linear regression results for the effect of air pollutants' interaction on visuo-construction

Variables	Coef.	Std.Err	t	*P*	95% Confidential interval	
					Lower	Upper
Total	-0.120	0.125	-0.970	0.334	-0.365	0.124
PM_1	-0.003	0.023	-0.130	0.899	-0.049	0.043
$PM_{2.5}$	0.003	0.015	0.190	0.849	-0.026	0.032
PM_{10}	-0.001	0.004	-0.250	0.801	-0.008	0.006
NO_2	-0.003	0.008	-0.360	0.717	-0.018	0.013
O_3	0.001	0.004	0.270	0.784	-0.007	0.009
Hypertension	-0.012	0.036	-0.340	0.733	-0.084	0.059
Stroke	-0.097	0.076	-1.280	0.199	-0.245	0.051
Smoke	-0.026	0.058	-0.450	0.655	-0.141	0.089
Social work	-0.007	0.015	-0.450	0.654	-0.036	0.023
Life event	0.044	0.046	0.950	0.341	-0.046	0.134
Child Death	0.025	0.039	0.650	0.513	-0.051	0.101

Note: Statistically significant results are bolded in the table ($P < 0.05$). Adjusted for hypertension, stroke, smoke, social work, life event and child death in each model. A phase lag has been set at the effect of air pollutants, a phase equals to two years. Total=lg(PM_1*$PM_{2.5}$*PM_{10}*NO_2*O_3)

Table S31 Panel data linear regression results for the effect of air pollutants' interaction on episodic memory

Variables	Coef.	Std.Err	t	*P*	95% Confidential interval	
					Lower	Upper
Total	-2.720	0.824	-3.300	**0.001**	-4.335	-1.104
PM_1	0.348	0.155	2.250	**0.024**	0.045	0.651
$PM_{2.5}$	-0.142	0.097	-1.470	0.142	-0.333	0.048
PM_{10}	0.020	0.024	0.830	0.406	-0.027	0.066
NO_2	0.054	0.052	1.030	0.305	-0.049	0.157
O_3	0.025	0.027	0.940	0.347	-0.027	0.078
Hypertension	-0.598	0.241	-2.490	**0.013**	-1.070	-0.126
Stroke	-1.533	0.500	-3.070	**0.002**	-2.513	-0.554
Smoke	-0.770	0.386	-1.990	**0.046**	-1.527	-0.013
Social work	0.185	0.099	1.870	0.062	-0.009	0.380
Life event	-0.140	0.303	-0.460	0.644	-0.733	0.453
Child Death	-0.337	0.256	-1.320	0.188	-0.839	0.165

Note: Statistically significant results are bolded in the table ($P < 0.05$). Adjusted for hypertension, stroke, smoke, social work, life event and child death in each model. A phase lag has been set at the effect of air pollutants, a phase equals to two years. Total=lg(PM1*PM2.5*PM10*NO2*O3)

Table S32 Panel data linear regression results for the effect of single air pollutant with White's Heteroskedasticity-Robust standard errors

Variables	PM_1				$PM_{2.5}$				PM_{10}				NO_2				O_3			
	Coef.	*P*	95% Confidential interval		Coef.	*P*	95% Confidential interval		Coef.	*P*	95% Confidential interval		Coef.	*P*	95% Confidential interval		Coef.	*P*	95% Confidential interval	
			Lower	Upper			Lower	Upper			Lower	Upper			Lower	Upper			Lower	Upper
Cognitive functioning	-0.093	**0.001**	-0.134	-0.053	-0.051	**0.001**	-0.071	-0.030	-0.030	**0.001**	-0.042	-0.017	-0.094	**0.006**	-0.161	-0.027	-0.039	0.113	-0.087	0.092
Attention	-0.014	0.070	-0.028	0.001	-0.007	0.086	-0.014	0.001	-0.003	0.188	-0.007	0.001	0.024	**0.038**	0.001	0.047	-0.005	0.517	-0.022	0.011
Orientation	-0.011	0.075	-0.023	0.001	-0.006	0.051	-0.012	0.000	-0.004	**0.018**	-0.008	-0.001	-0.004	0.664	-0.022	0.014	-0.006	0.338	-0.019	0.007
Visuo-construction	-0.010	**0.001**	-0.016	-0.005	-0.005	**0.001**	-0.008	-0.002	-0.003	**0.001**	-0.005	-0.002	-0.014	**0.002**	-0.022	-0.005	-0.005	0.137	-0.011	0.001
Episodic memory	-0.059	**0.001**	-0.093	-0.024	-0.033	**0.001**	-0.050	-0.015	-0.019	**0.001**	-0.030	-0.009	-0.101	**0.001**	-0.159	-0.043	-0.022	0.283	-0.063	0.018

Note: Statistically significant results are bolded in the table ($P < 0.05$). Adjusted for hypertension, stroke, smoke, social work, life event and child death in each model.

Table S33 Panel data linear regression results for the interaction of air pollutants with White's Heteroskedasticity-Robust standard errors

Variables	Coef.	Std.Err	t	*P*	95% Confidential interval	
					Lower	Upper
Cognitive functioning	-2.820	1.000	-2.820	**0.005**	-4.781	-0.859
Attention	0.393	0.347	1.130	0.257	-0.287	1.072
Orientation	-0.373	0.258	-1.450	0.148	-0.878	0.133
Visuo-construction	-0.120	0.130	-0.920	0.355	-0.376	0.135
Episodic memory	-2.720	0.844	-3.220	**0.001**	-4.375	-1.064

Note: Statistically significant results are bolded in the table ($P < 0.05$). Adjusted for hypertension, stroke, smoke, social work, life event and child death in each model. Total=lg(PM_1*$PM_{2.5}$*PM_{10}*NO_2*O_3)

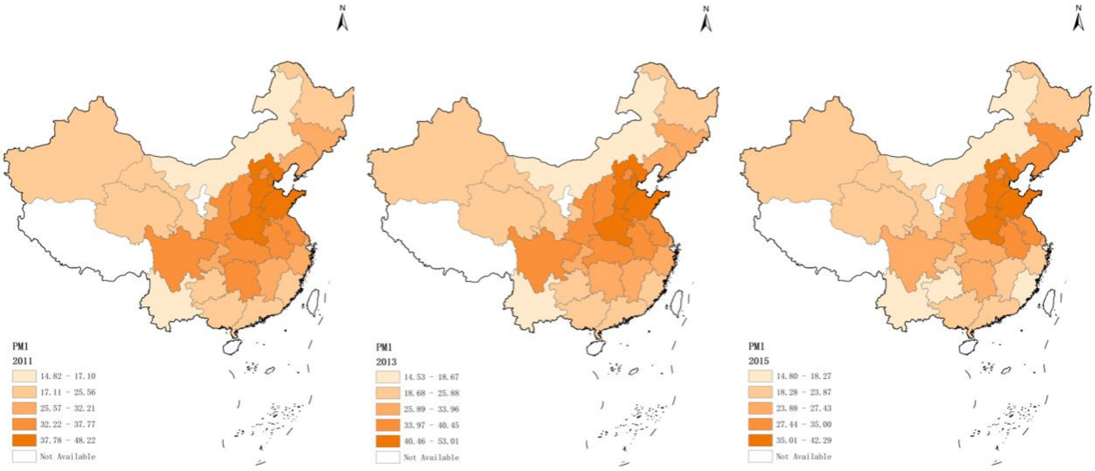

a. b. c.

Fig. S1. The spatial distribution of average PM_1 concentration
a. wave 1, b. wave 2, c. wave 3.

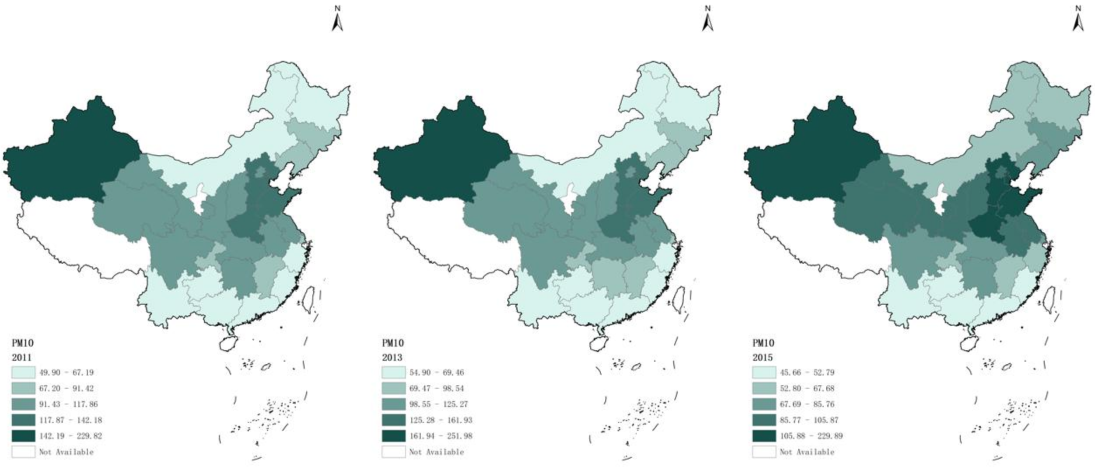

a. b. c.

Fig. S2. The spatial distribution of average PM_{10} concentration
a. wave 1, b. wave 2, c. wave 3.

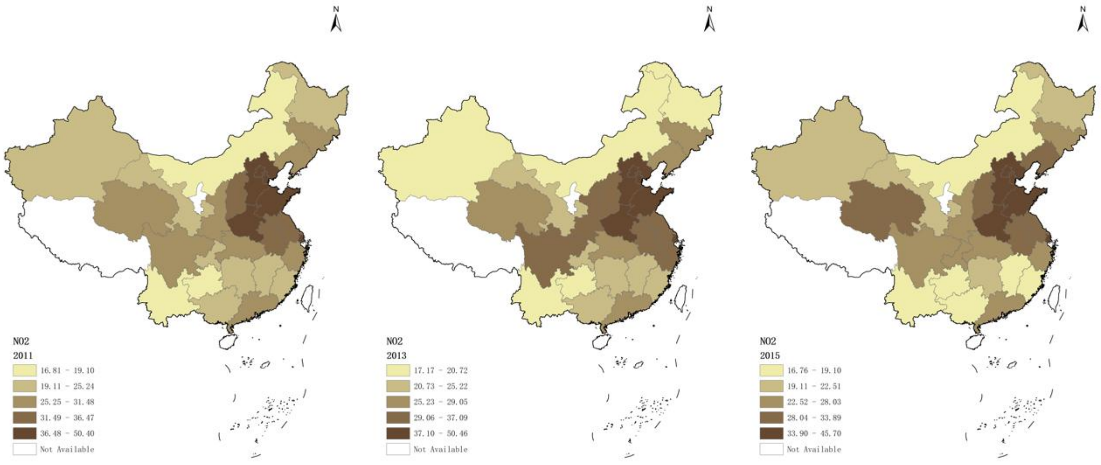

a. b. c.

Fig. S3. The spatial distribution of average NO_2 concentration
a. wave 1, b. wave 2, c. wave 3.

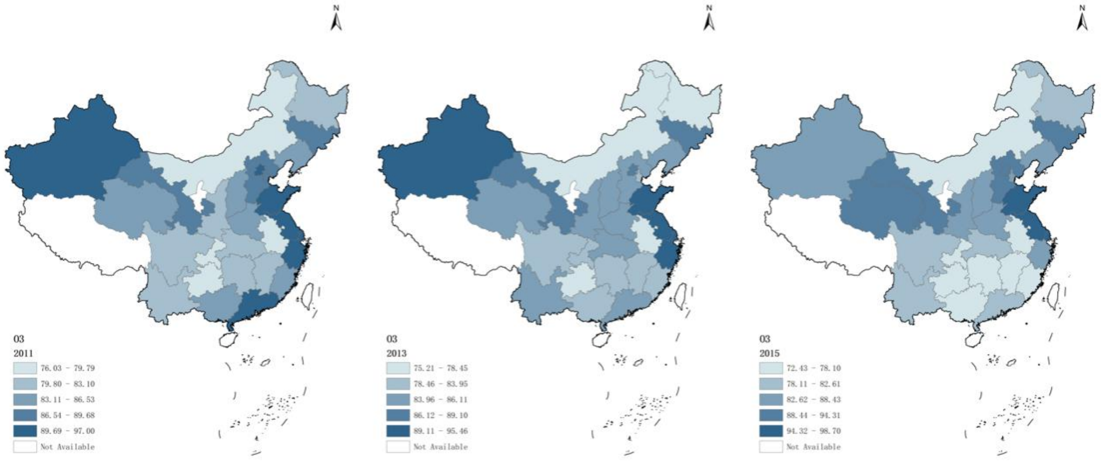

a. b. c.

Fig. S4. The spatial distribution of average O_3 concentration
a. wave 1, b. wave 2, c. wave 3.

Abbreviation list

DBP Diastolic Blood Pressure

PM_1 Particulate matter with aerodynamic diameters ⩽ 1μm

$PM_{2.5}$ Particulate matter with aerodynamic diameters ⩽ 2.5μm

PM_{10} Particulate matter with aerodynamic diameters ⩽ 10μm

NO_2 Nitrogen dioxide

O_3 Ozone

BMI Body mass index

CAAA Clean Air Act Amendments

CAPI Computer Assisted Personal Interviewing

CFPS China Family Panel Studies

CHAP China High Pollutant dataset

CHARLS China Health and Retirement Longitudinal Study

CI Confidential interval

CLHLS China Health and Retirement Longitudinal Study

Coef. Coefficient

IQR Inter-quartile range

K-W test Kruskal-Wallis Rank sum test

MCI Mild cognitive impairment

MMSE Mini-Mental State Examination

OLS Ordinary least squares

SBP Systolic Blood Pressure

SMS Sample Management System

WHIMS Women's Health Initiative Memory Study

WHO World Health Organization

YLL Years of life lost
